# There’s education, and then there’s education in medicine

**Published:** 2016-07

**Authors:** KENNETH D. ROYAL, JASON C.B. RINALDO

**Affiliations:** 1 Department of Clinical Sciences, North Carolina State University, Raleigh, NC, USA;; 2 Department of Assessment and Instructor, Rawls College of Business, Texas Tech University, Lubbock, TX, USA

**Keywords:** Medical education, Education, Medicine, Education research, Quality, Research design

## Abstract

**Introduction:**

For some time now the field of medical education has been criticized by many of its stakeholders. Countless debates have been presented in the literature regarding the quality of medical education research, adequacy of methodological rigor, and other concerns.

**Methods:**

At present, the views expressed have largely come from physicians and individuals with less familiarity with education science.

**Results:**

As prolific educational researchers with Ph.Ds in Education and Psychology, we offer a critique of medical education's apparent identity crisis and address what we believe are some of the most significant problems continuing to impede the field of medical education from catching up with the broader field of education. We close with specific recommendations for improving the overall state of medical education.

**Conclusion:**

Finally, both editors and reviewers for medical education journals need to abandon the hegemonic views regarding research design. Thus, research designs that many in the clinical sciences often perceive as ‘weak’ are entirely appropriate in education research fields.

## Introduction

For some time now the field of medical education has been criticized by many of its stakeholders. Countless debates have been presented in the literature regarding the quality of medical education research, adequacy of methodological rigor, and other concerns (1). Similarly, a number of researchers have also questioned how medical education compares to mainstream education fields (2). Although these topics have been widely discussed in the literature, the views expressed have largely come from individuals who focus heavily on medicine, and less on education. As prolific educational researchers with Ph.Ds in Education and Psychology, we offer a critique of medical education’s apparent identity crisis and address what we believe are some of the most significant problems continuing to impede the field of medical education from catching up with the broader field of education. We believe these serious issues cannot be dealt with unless confronted head-on, so we wish to apologize in advance if we offend some readers.

**There’s education, and then there’s education in medicine**

First, anyone in the medical field that is involved in education in any capacity can be considered a “medical educator”. Thus, medical educators vary considerably in terms of their experience, educational backgrounds, disciplinary training, etc. The vast majority of medical educators are physicians with limited formal training in the education field, despite extensive exposure to education. Within medicine, it is accepted that having been to a physician many times does not tend to confer medical knowledge or expertise to the patient. Similarly, within education, having been in many classrooms does not make a person expert in education (higher education faculty notwithstanding). Alongside the ‘experientially trained’ medical educators who have sat through many classes, there are pockets of medical educators who were formally trained in teaching methodology and scientific principles of learning. The extreme variability in skill and training among medical educators is something that presents a source of confusion. The relative scarcity of those who have thoroughly studied the scientific literature of educational methodology within the field of medical education makes it difficult for most medical educators to effectively discern ‘good’ ideas from ‘poorer’ ideas when evaluating the quality of educational techniques and educational research methods.

Second, as individuals formally trained in education science and human cognition, we recognize that education is a broad discipline all its own. This view appears to be in stark contrast with most medical educators who view education as a sub-discipline of medicine. In our view, this is most problematic. While we certainly recognize the necessity of education scholarship geared specifically toward medicine, the isolation from the mainstream education community has continually cost the field of medical education in terms of knowledge production, innovation, and financial resources. For example, many of the problems medical educators are identifying today and attempting to solve were identified and solved decades ago by educational researchers who devoted their careers to studying such issues. Ignoring their work is inefficient, ineffective, and reflects the lack of exposure to the scientific study of learning and teaching. We are unsure exactly why this divide exists, whether intentional due to inaccessibility of educational language and concepts (or some other reason), or more incidental, but the fact is that this disconnect has repeatedly been evidenced to have a very detrimental effect on medical education. To state another way, simply being smart and thinking hard about educational challenges is an inadequate substitute for knowledge of 80 years of education research. Believing it is a substitute is a major cognitive error.

Third, there remains a great deal of confusion about where to find educational research. In our view, those medical educators with formal training in education fields must bear the responsibility for this failure. After all, persons without formal training in education should not be expected to know this. What we typically observe is because physicians have a great deal of familiarity with PubMed, this becomes the default search engine for educational literature. Because some educational research is indeed indexed in PubMed, that provides a red herring to an unknowing physician. It is searching at night under the streetlamp for one’s lost keys, because that is what can be seen. However, it should be made abundantly clear that an education researcher will rarely, if ever, resort to PubMed for educational research. PubMed is a biomedical repository, not an educational repository. To find educational research, one should solicit databases such as EBSCO, ERIC, PsycInfo, ProQuest, and JSTOR among many others. PubMed is among the last places where an educational researcher would look for educational literature. This perpetual misunderstanding can no longer go ignored. To illustrate this disconnect, consider this simple example:

If an education researcher sought treatment guidelines for home management of an upper respiratory infection for her children, it would be a very poor plan to search the vast but familiar education literature for such guidelines. Instead, she would look in medicine, within the field originating that knowledge. Similarly, if a physician in a teaching role is seeking effective management of the education and classroom of her students, it would be a poor plan to search the vast but familiar medical literature for guidelines on effective teaching.

 Fourth, there are a number of journals that focus on medical education content. However, those responsible for these journals must also assume some responsibility for medical education being conceived as a narrow field. If one picks up the most leading medical education journals, it will become immediately clear that the field is both insular and recursive, as the overwhelming majority of articles are plagued with citations limited to the confines of the medical education arena. The field of medical education cannot properly grow and advance if it operates in a silo. To be clear, educational thought-leaders do not typically publish in medical education journals, and there is an enormous amount of rich information to be learned from education experts outside the confines of medical education. While we recognize that searching outside the medical education literature may create an unwelcome challenge for many medical educators, the costs of failing to do so only creates more challenges and hindrances. Thus, an effective auxiliary role for medical education journals is to provide an intellectual bridge between the vast educational literature and the peculiar application of educational methods in medicine.

This also leads to another issue that is incredibly common, and that involves the notion of ‘peer review’ in medical education journals. A great deal of frustration with peer review in medical education journals arises from reviewers with medical rather than educational expertise. Most medical education journals consist primarily of physicians serving as peer reviewers of medical education content.  This is problematic for several reasons. Although physicians can speak to the appropriateness of the medical content and perhaps various contextual factors, as mentioned before, they are typically unaware of educational science. Physicians are less likely to be well-versed in the education literature outside the confines of medical education, and consequently, are less able to distinguish an innovation from a previously tested educational idea or approach. As a case in point, in recent years the notion of a “flipped classroom” has been all the rage. However, most medical educators are shocked to learn the flipped classroom model has been around for over 100 years and is a mundane model that many disciplines, including the social sciences, humanities, education, and business, use every day in colleges and universities everywhere (3).

Similarly, educational researchers tend to be frustrated with expectations of experimental or quasi-experimental designs. The fact that many, if not most, medical education journals expect educational research studies to incorporate some form of an experimental or quasi-experimental design is proof that they are generally unfamiliar with educational research and ethics constraints. Educational research rarely utilizes experimental or even quasi-experimental designs, and when such rare studies do surface, they are almost always conducted by persons outside the education field (4). A review of dissertations completed in the field of education revealed less than 1% involved randomized experiments (5). Education is not alone, as fields such as sociology, political science, macroeconomics and management also rarely utilize randomized experiments (6). Educational researchers have long recognized that experimental designs generally are neither appropriate, nor effective for educational research. Cook says “Random assignment is not politically, administratively, or ethically feasible in education” (4). The very fact that medical education scholars continue to debate the appropriate treatment of educational data today, an issue that was long ago resolved in education, is symptomatic of just how far behind the field of medical education is from other education fields.

To be clear, educational research studies that lack some form of an experimental (or quasi-experimental) design are not ‘flawed’, ‘weak’, or otherwise incapable of producing valid (and useful) findings. The use of non-experimental designs, convenience samples, etc.  is very much the convention of the field given the types of data with which educational researchers work. Further, Institutional Review Boards (IRBs) deem unethical any research study that withholds an educational benefit from students. Even the notion of a placebo control, which may be entirely appropriate in medicine, typically does not translate well to education as the use of a placebo could essentially have an effect on students that delays their education, development, and otherwise incurs a number of other costs. Thus, physicians make a terrible mistake when they fail to understand the patient/student parameters and ethics and attempt to promote a ‘superiority of science’ position in medical education. While it is understandable that physicians tend to promote what they know (clinical research), one must bear in mind that when venturing into educational research, one is making a departure from medicine and a voyage into the field of education. This transition from one disciplinary field to another should involve a reassessment of one’s assumptions for a wise physician. Deeply troubling, though, is the fact that some medical education journals explicitly state that they will not consider publishing a paper unless it involves some form of experiment. Contrary to these editors’ beliefs, such uninformed views, divisive language, and ill-guided perspectives are not helping the field of medical education.

**Recommendations for improving the state of medical education**

To this point, we have pointed out what we perceive as being some of the most critical issues surrounding medical education’s relatively slow growth as compared to mainstream education. Now, we will present what we believe are some potential solutions to the aforementioned problems.

First, there is a significant shortage of medical educators with formal training in education. Individuals with formal training in education should be among the field’s most visible and most vocal, as these individuals typically already possess the knowledge and skill bases to immediately become thought-leaders in the field. At present, this is not the case. Many of these individuals remain marginalized by tired debates about experimental designs and uninformed methods commentary regarding incompatible research designs typically used in the clinical sciences, or simply a lack of collegial awareness and understanding of what these individuals know, or do, and what they can contribute to a medical program. Until a shift occurs in which medical education is sufficiently populated with education professionals the entire medical education enterprise will continue to operate at less than optimal capacity.

Second, the field of medical education must abandon its insular practices and begin to grow closer to the body of scientific literature in education. Medical educators should routinely survey the education literature and attend education research conferences when possible. Many will be surprised at both how inexpensive and rewarding these conferences typically are. While it is certainly true that many medical educators will find much of the language used in mainstream education inaccessible at first, those persistent will quickly climb the learning curve and become more fluent. Those that commit themselves to educational research will find they will have much more to contribute to medical education than previously realized. A good practice would be for those working in medical schools to reach out to individuals working in colleges of education and develop collaborative opportunities (e.g., take part in seminars, brown bag luncheons, attend research conferences held on one’s campus, and so on). 

Third, we must do a better job communicating where one should search for educational literature. As noted previously, PubMed is not an appropriate resource for obtaining educational literature. In fact, the education-related material appearing in PubMed is only a very small fraction of the larger body of educational research available. Medical educators must familiarize themselves with the databases specifically dedicated to educational research. If unsure which databases your institution has access to, most library staff are incredibly helpful and will be happy to provide a customized workshop for your department/team in which they present a variety of options for locating and accessing educational literature.

Fourth, editors of medical education journals should seek to place considerably more individuals with formal educational training on their editorial and review boards. These individuals are likely to be better versed at education, and the results will likely show in the quality of feedback provided to authors, and ultimately, the quality of published materials. Further, these individuals will likely be instrumental in ensuring a more fair review, offering more diverse perspectives, and offering invaluable feedback to editors soliciting feedback for resolving a dispute. To be clear, there is no shortage of competent educational researchers. Many will be delighted to offer their services for reviewing manuscripts; however, they must first be asked.

Hostetlersays “Good [educational] research is a matter not only of sound procedures but also of beneficial aims and results” (7). In medicine, correlational studies are routinely published that identify associations between risk factors and a disease. These studies, despite their many limitations, are generally both incredibly valuable and well-received by others in the field. Does it not seem pedantic to insist upon double blind placebo control in educational studies with students, especially given all their psychological complexities, when even the field of medicine seems to accept the value and utility of non-experimental studies? A number of excellent texts exist for individuals looking for guidance on how to properly conduct educational research. Medical educators should see Creswell, McMillan and McMillan and Schumacher to name a few (8-10).

## Conclusion

Finally, both editors and reviewers for medical education journals need to abandon the hegemonic views regarding research design. Thus, research designs that many in the clinical sciences often perceive as ‘weak’ are entirely appropriate in education research fields. To view educational research designs as inferior to clinical research demonstrates ignorance of educational research and ethics. 
